# Quantification of Colistin in Plasma by Liquid Chromatography-Tandem Mass Spectrometry: Application to a Pharmacokinetic Study

**DOI:** 10.1038/s41598-020-65041-w

**Published:** 2020-05-18

**Authors:** Kamal M. Matar, Batool Al-Refai

**Affiliations:** Department of Pharmacology & Therapeutics, Faculty of Pharmacy, Kuwait University, Kuwait, Kuwait

**Keywords:** Chemical biology, Antimicrobials

## Abstract

Colistin is a polymixin antibiotic (polymixin E) that is produced by *Bacillus colistinus* bacteria. The aim of the present study was to develop and validate a method to quantify colistin levels in plasma using high performance liquid chromatography-tandem mass spectrometry (LC-MS/MS) technique and then apply it in experimental animals (rats) to investigate the pharmacokinetic profile of colistin in this species. Polymyxin B was used as an internal standard (IS) and the quantitation was carried out using ESI + interface and employing multiple reaction monitoring (MRM) mode. A mobile phase consisting of acetonitrile:water:formic acid (30:70:0.1%; *v/v/v*) was employed and Zorbax eclipse plus C_18_ (1.8 µm, 2.1 mm i.d. x 50 mm) was the optimal column for this method and utilized at a flow rate of 0.2 mL/min. The full scan mass spectra of precursor/product ions of colistin A were at *m/z* 585.5 > 100.8, for colistin B at *m/z* 578.8 > 101 and for the IS at *m/z* 602.8 > 101. The lower limit of quantification (LLOQ) was 0.5 µg/mL. The method demonstrated acceptable intra-run and inter-run precision and accuracy for both colistin A and colistin B. Colistin was stable when assessed for long-term stability, freeze-thaw stability and autosampler stability. However, it was not stable when stored at room temperature. The matrix effect evaluation showed minimal or no effect. Incurred sample reanalysis findings were within acceptable ranges (<20% of the nominal concentration). The pharmacokinetic parameters of colistin were investigated in rats using the present method. The developed method for colistin demonstrates that it is rapid, sensitive, specific, accurate, precise, and reliable.

## Introduction

Resistance to antibiotics is an ongoing concern of the medical and pharmaceutical industries. The increased resistance of Gram-negative bacteria and the limited number of sensitive antibiotics in the market has made their resistance an issue that is being addressed worldwide^1,2^. Cases of chronic pulmonary infections with *Pseudomonas aeruginosa* (*P. aeruginosa)* are an immense challenge to health care professionals when dealing with cystic fibrosis (CF) patients^3^. This type of infection is also a public health dilemma^4^. The increase in the number of cases infected with multi-drug resistant (MDR) Gram-negative bacteria has led to the re-use of polymixin antibiotics^5^. These MDR Gram-negative bacteria are often of the following species: *P. aeruginosa, Acinetobacter baumannii (A. baumannii)* and *Klebsiella pneumoniae (K. pneumoniae)* which developed strains that are mostly susceptible only to polymixins^6–8^.

Microbiologically, colistin is a polymixin antibiotic (polymixin E) that is produced by *Bacillus colistinus* bacteria^9^. Polymixins were discovered in 1947^10,11^, while ‘colistin’ was isolated by Koyanna and colleagues in 1950^9^ and introduced in the market in 1959^12^.

**Figure 1:**
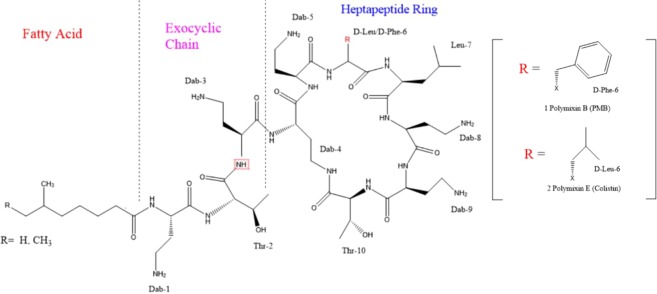
Chemical structures of colistin and polymixin B.

Polymixins are cyclic polypeptides antibiotics. Five polymixins (A to E) were produced by the soil bacterium *Bacillus polymyxa* and were identified in the 1940’s^10,11^. Out of these compounds, only two have been recognized to possess antibacterial activity; polymixin B (PMB) and polymixin E (colistin)^13^. These two compounds share the same primary sequence and are secondary metabolite nonribosomal peptides (Fig. 1).

Colistin is recently used against bacteremia and ventilator-associated-pneumonia (VAP) which are caused by *P. aeruginosa*^13,14^. The use of inhaled colistimethate sodium (CMS) in intensive care unit patients for the treatment of VAP has been reported^15,16^. Colistin has proven effective when no other treatment is available for the treatment of multi-resistant bacteria in major burn patients^17^. Intraventricular administration of colistin has recently been used for the treatment of ventriculitis that is caused by MDR *A. baumannii*^18,19^.

Following an oral administration, colistin sulphate is poorly absorbed from the gastrointestinal tract (GIT)^20^. However, colistin sulphate has been reported to be well absorbed after an oral administration in infants^12^. On the other hand, it has been reported that the inhalation of colistin sulphate in cystic fibrosis (CF) patients resulted in a maximum serum concentration of 0.13 mg/L after 1.5 h^21^. Although CMS is rapidly absorbed after an intramuscular injection in humans^22^, it is not absorbed through burns, inflamed skin or mucosal membranes^23^ and is poorly absorbed through the GIT^24^. The V_d_ of CMS is limited because of its polarity and high molecular weight^25^. Following an IV dose of CMS (1.63–3.11 mg/kg every 8 h) in CF patients, the V_d_ was 0.34 ± 0.10 L/kg at steady state^26^. The renal route is the main elimination pathway of CMS and it has a t_1/2_ of 124 ± 52 min in CF patients^26^ in contrast to colistin which is eliminated extra-renally and its elimination half-life (3 h) is longer than that of CMS^27^.

Several techniques have been reported to quantify colistin in plasma^28–34^. Of these, microbiological assays which were used to measure colistin levels in clinical settings and their use was limited because of their non-specificity^30^. Chromatographic methods were also reported for colistin quantification in biological samples. The limitations of HPLC procedures involve longer analytical run times, larger sample volumes requirement as well as inadequate lower limit of quantification (LLOQ)^28,32^. On the other hand, liquid chromatography coupled to mass spectrometry (LC-MS/MS) is more accurate, precise, sensitive, and selective technique. However, some of the previously reported LC-MS/MS assays were lacking internal standards (IS)^29^, used high flow rates (1.0 ml/min)^33^, did not assess matrix effect^34^, needed high-cost sample pretreatment procedures or utilized selected ion monitoring (SIM) instead of multiple reaction monitoring (MRM) modes^29^.

The objectives of the present study were to develop and validate a simple, rapid and reliable LC-MS/MS method for quantification of colistin in plasma. In addition, to apply the developed method to estimate the pharmacokinetic parameters of colistin in rats.

## Experimental

### Chemicals and materials

Colistin sulfate, polymixin B (PMB) sulfate, trichloroacetic acid (TCA), hydrogen peroxide solution 30% (w/w) in water, isopropyl alcohol and formic acid were all purchased from Sigma-Aldrich Co. (St. Louis, MO, USA). Acetonitrile (LC-MS grade) was purchased from Fisher Scientific International (Fairlawn, NJ, USA) and methanol (LC-MS grade) was purchased from VWR (Radnor, PA, USA). All chemicals and reagents were of high purity. Oasis HLB 1cc Vac cartridges, 30 mg sorbent per cartridge, 30 µm particle size (Oasis® HLB 1 cc, 30 mg), (Waters Assoc., Milford, MA, U.S.A.) were used in solid phase extraction procedures.

### Instrumentation and chromatographic conditions

The LC-tandem mass spectrometry (LC-MS/MS) system used was composed of separation module (Alliance e2695), an autosampler and solvent delivery system, as well as Micromass Quattro Micro-Electrospray ionization (ESI) triple-quadrupole tandem mass spectrometer (Waters Assoc., Milford, MA, U.S.A.). Analytical separation of the analytes and the IS was performed on Zorbax eclipse plus C_18_ column (1.8 µm, 2.1 mm i.d X 50 mm) (Agilent Technologies, Santa Clara, CA, USA). The mobile phase (acetonitrile:water: formic acid; 30:70:0.1, *v/v/v*) was delivered at a flow rate of 0.2 ml/min. Data acquisition was performed using MassLynx software (v. 4.1, Micromass, Manchester, UK).

MS and MSMS tuning parameters were optimized for both colistin and IS (polymixin B; PMB) by direct infusion of colistin and IS solutions in the mobile phase at a concentration of 10 µg/ml each into the ionization probe using a Hamilton syringe (flow rate = 10 µl/min). The optimized tuning parameters were: capillary voltage = 3.60 kV, cone voltage = 30 V, source temperature = 150 °C, desolvation temperature = 450 °C, collision energy = 30 eV and dwell time = 0.2 sec. The MS-MS conditions were also optimized throughout the development of the assay for colistin and the IS and were operated in a positive electrospray ionization (+ESI) interface. Additionally, the transition of the precursor to the product ions for the analytes and IS were used for obtaining MRM.

### Preparation of solutions

Colistin sulfate was prepared by dissolving 5.5 mg of the powder in 5 ml water to make a stock solution of 1 mg/ml of colistin. PMB sulfate solution of 1 mg/ml was also prepared by dissolving 5.8 mg of PMB sulfate powder in 5 ml water. Both solutions were stored at −70 °C. Calibration standards and quality controls were prepared by spiking different amounts of colistin sulfate stock solution in plasma. Aliquots of colistin sulfate were further diluted to provide standard concentrations of 0.5, 1, 2, 5, 10, 15, and 20 μg/mL (equivalent to: 78, 156, 312, 780, 1560, 2340 and 3120 ng/ml of colistin A and 353, 706, 1412, 3350, 7060, 10590 and 14120 ng/ml of colistin B) whereas, the concentrations of 1.5, 3.5, 7.5 and 17.5 μg/mL (which contained 234, 546, 1170 and 2730 ng/ml of colistin A and 1059, 2471, 5295 and 12355 ng/ml of colistin B) were considered as quality controls. The calibration standards and quality controls were aliquoted (150 μL) into 0.5 ml Eppendorf tubes and stored at −70 °C pending analysis.

### Sample pre-treatment

To 100 μl rat plasma sample, 5 μl of 500 μg/ml IS was added and followed by addition of 400 μl of 50:50 (*v/v*) methanol-TCA (10%). The samples were vortex-mixed for 1 min, rota-mixed for 10 min at 40 rpm and then centrifuged at 12,000 rpm for 10 min. Following centrifugation, 400 µl of the supernatant was collected and re-extracted with solid phase extraction (SPE) using SPE (Oasis HLB 1 cc, 30 mg) cartridges. First, the cartridges were conditioned with 500 μl of methanol then 500 μl of water. After that, 400 μl of the supernatant was transferred and then washed with 500 μl of water. The tubes were changed and then the samples were eluted with 150 μl of methanol: water: formic acid (80:19:1%, *v/v/v*) solution. The elution step was repeated twice, and then 250 μl of the solution was transferred to the low volume inserts and 20 μl of this solution was injected into the LC-MS system.

### Validation of the assay method

The present assay method was fully validated according to the international standards^35^. In this regard, the validation parameters including linearity, LLOQ, selectivity, accuracy, precision, matrix effect, injection carry-over, incurred sample reanalysis (ISR), and stability studies were investigated.

### Linearity

The linearity of the calibration curve is an important parameter that demonstrates the relationship between the instrument response and the known concentration of the analyte. The standard curve was constructed using a drug-free plasma sample, zero standard, and seven non-zero plasma standards covering the concentrations expected in the study. The original concentrations were then plotted against the responses to obtain the slope, intercept and correlation coefficient (r^2^) by the least-square linear regression methods using MassLynx software (v. 4.1, Micromass, Manchester, UK). The precision and accuracy results shouldn’t deviate by more than 15% (RSD,% ≤ 15%) except at the LLOQ where the values should not deviate by more than 20%.

### Selectivity

Selectivity is the ability of the method to distinguish the targeted analyte from other substances present in the sample, and it was investigated by analyzing six different sources of drug-free (blank) rat and human plasma samples for potential interferences and this was compared with the spiked plasma samples at the LLOQ. The mass detector responses (peak area) of the different plasma extracts (at the retention times of colistin A, colistin B and IS) were compared to that of the spiked blank rat and human plasma samples at the LLOQ.

### Accuracy and precision

Accuracy is the closeness of the measured value to the true concentration and it was determined by analyzing analyte samples of known concentrations. In this regard, four concentrations (quality controls; QC) covering low, medium and high range of the calibration curve within the therapeutic range of colistin were investigated with six replicates for each concentration. Precision determines the closeness of the obtained results for an individual quality control sample to each other when using the developed method to analyze different aliquots of the sample. Accuracy (bias,%) was measured as the percent of deviation from the nominal concentration whereas, the precision was determined as the relative standard deviation from the mean (RSD,%). The accuracy and precision of the QCs should be ≤15%.

### Stability tests

Various types of stability tests including freeze-thaw stability and long-term stability were assessed. The freeze-thaw stability test was performed by removing the four QC samples out of the storage temperature (−70 °C) and keeping them at room temperature (25 °C) until thawing and then aliquots of the samples were analyzed. After that, the samples were again returned back for freezing in the same storage conditions for 12 to 24 h. The freeze-thaw cycles were repeated five times and 4 replicates of the analytes were assayed. In addition, a long-term stability test was performed to ensure that the stability of the samples for a time that exceeds the interval between the first sample collection and the last sample analysis. Four aliquots of low, medium and high concentrations (QC) were kept for 20 days in the same temperature used to store samples regularly at the lab (−70 °C) then analyzed and compared to the nominal concentration of freshly prepared QC samples. Moreover, the stability in the autosampler (4 °C) was assessed by keeping the processed samples under this condition and assayed periodically at different times for up to 24 hours. In addition, benchtop stability test was performed by keeping the samples at room temperature (25 °C) for six hours and analyzing them during this time. The mean value results of stability tests were calculated and compared to the nominal concentrations.

### Matrix effect

The effect of all endogenous components existing in the sample other than the analyte of interest is known as the matrix effect, and it affects the selectivity of the method by causing ion suppression or ion enhancement. This means that the co-eluting endogenous compounds affect the ionization of the analyte. Thus, the effect of the matrix is vital and should be assessed. In evaluating the effect of the matrix, spiked samples prior to extraction, spiked samples after extraction and aqueous samples were prepared using the four QC concentrations. Matrix from 6 different lots of different sources of plasma samples was used and it was studied at low, medium and high levels of calibration curve. The matrix effect was assessed using Matuszewski method^36^.

### Injection carryover

Carryover can occur when injecting different samples in a row, particularly after injecting samples with high concentrations followed by low concentration samples. This effect was studied by injecting high concentration calibration standard or the upper limit of quantification (ULOQ) (20 µg/ml) followed by drug-free (blank) plasma samples. The test was conducted four times and the results were consequently assessed. The resulting response should not be more than 20% of the LLOQ of colistin A and colistin B or 5% for the IS.

### Incurred sample reanalysis

Incurred sample reanalysis (ISR) was assessed by re-analysis of some rat plasma samples^37^. The original and repeated analyses of rat samples were achieved using the present method. The % difference of the original and repeated colistin A and colistin B concentrations was determined using the following formula:

$$ \% \,{\rm{Difference}}=100\ast ({\rm{Repeated}}-{\rm{Original}})/{\rm{Mean}}$$

### Pharmacokinetic study

The present study was approved by Ethical Committee of Health Science Center- Kuwait University. Male Sprague Dawley^®^ (MSD) rats (21 weeks ± 3 days of age) with a body weight of 450–550 g [supplied by the Animal Resource Center (ARC), Health Sciences Center (HSC), Kuwait University] were used to estimate the pharmacokinetic profile of colistin. The animals (n = 8) were housed in 12 h/12 h light/dark cycle. The animals had free access to standard laboratory rat chow (Labdiet™ #5012) as well as tap water. All the animals were subjected to left jugular vein cannulation, IV drug administration, and subsequent blood sampling. All surgical procedures were carried out under strict aseptic conditions. In addition, all surgical instruments and materials were autoclaved, all solutions were sterile and the surgical procedure was performed with the aid of surgical microscope (Leica wild M690). Animal body temperatures were maintained around 37.6 ^°^C (rectal) by placing the animals on an electronic heating pad throughout the surgical procedure as well as the post-surgical recovery period. About 350 µl of rat blood sample was collected from each animal. From this sample, 50 µl of blood sample was transferred to a capillary tube, sealed with the sealant clay at one end and centrifuged to calculate the Packed Cell Volume (PCV); i.e., Hematocrit. The remaining rat blood sample (300 µl) was centrifuged at 9000 *X* g for 10 min and aliquots of 100 µl of plasma sample was collected and stored at −70 °C pending analysis. All methods described have been conducted following Good Laboratory Practice guidelines.

Colistin was quantified in the biological samples by the present LC-MS/MS method and then the pharmacokinetic parameters (t_1/2_, CL, V_d_, AUC) were estimated by non-compartmental method employing Kinetica^®^ software, version 5.1 (Thermo Fisher Scientific, Waltham, USA).

## Results and Discussion

### LC-Mass spectrometry

**Figure 2:**
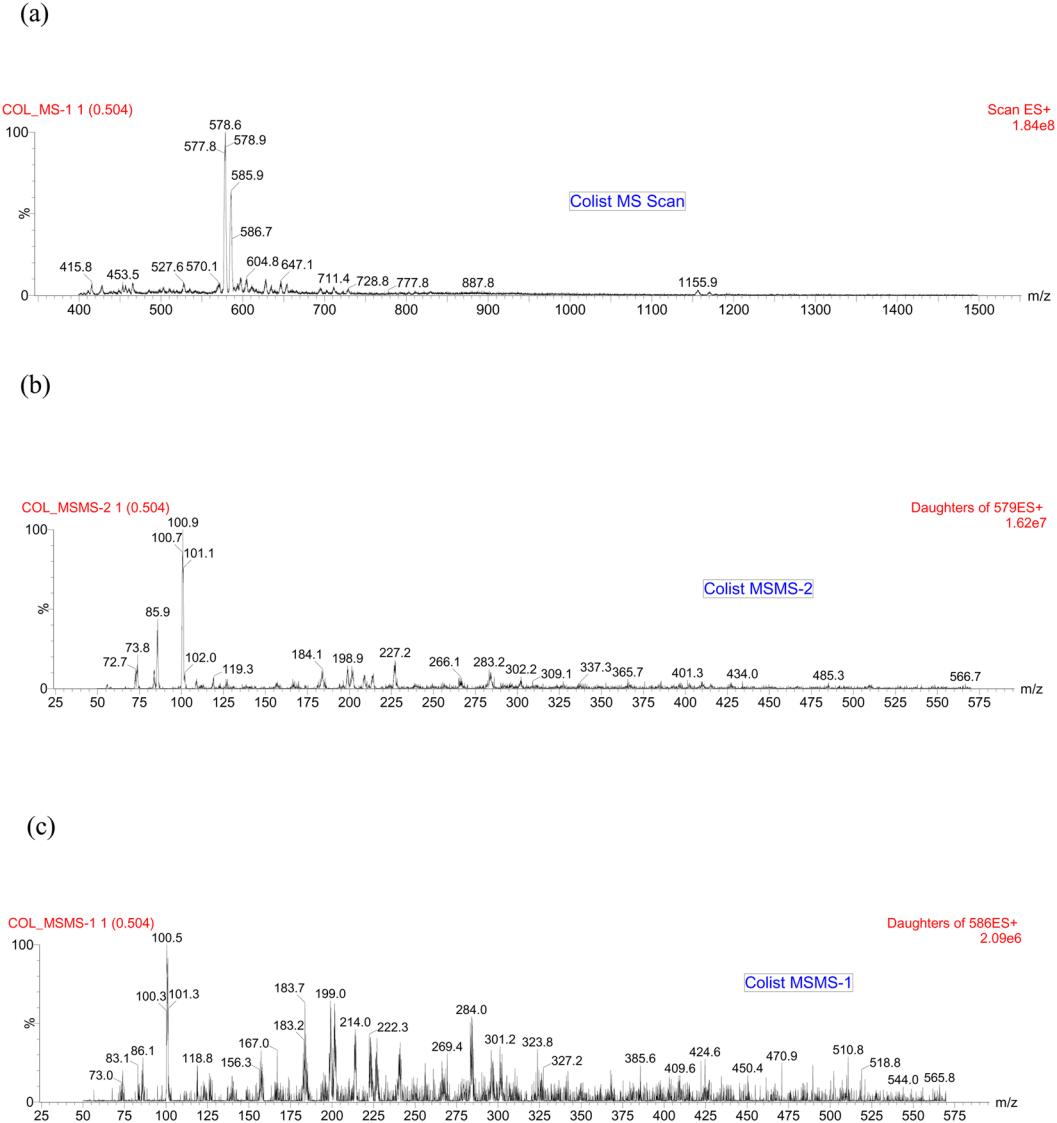
Full scan mass spectra of (**a**) precursor ions of colistin A and colistin B, (**b**) product ions of colistin A and (**c**) colistin B.

**Figure 3:**
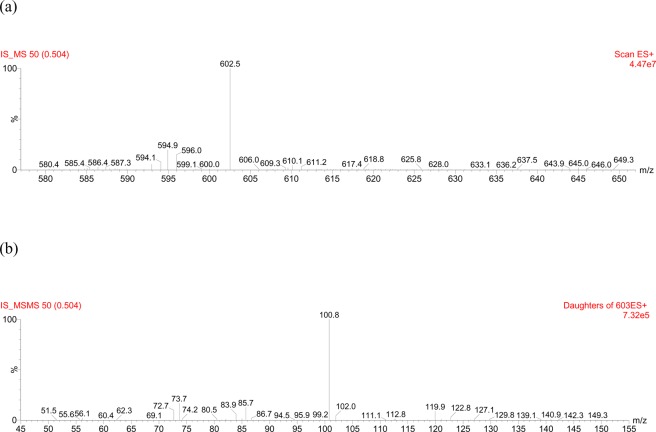
Full scan mass spectra of (**a**) precursor ion and (**b**) product ions of IS (PMB).

**Figure 4:**
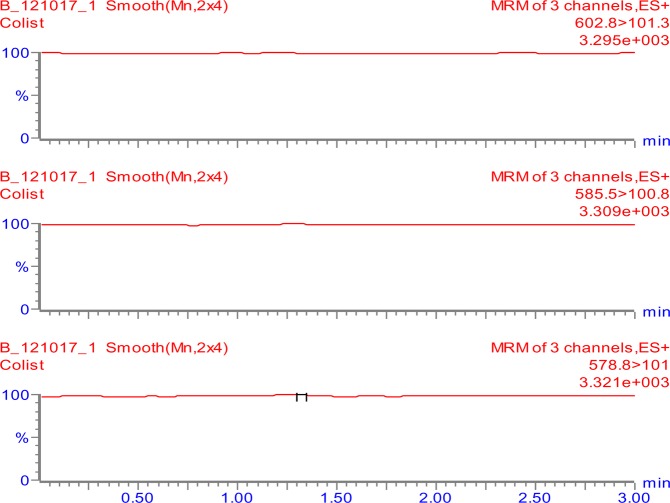
Typical LC-MS/MS-MRM chromatograms of a drug-free rat plasma.

**Figure 5:**
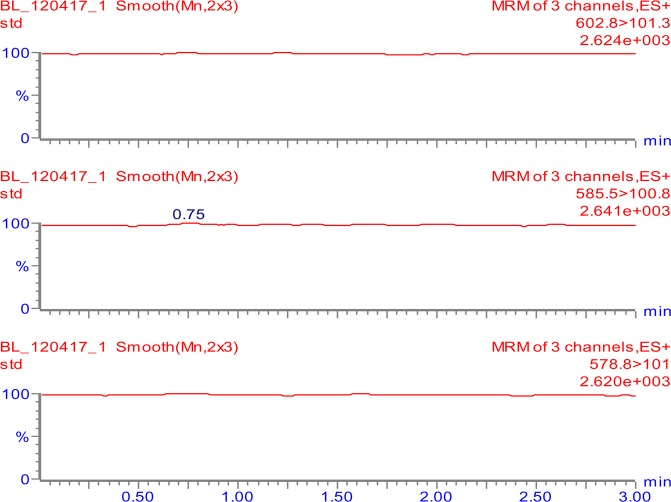
Typical LC-MS/MS-MRM chromatograms of a drug-free human plasma.

**Figure 6:**
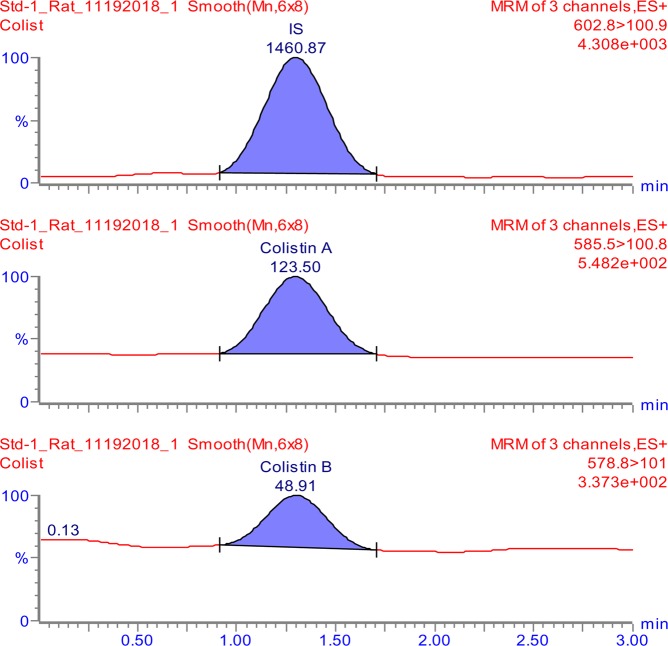
Typical LC-MS/MS-MRM chromatograms of a drug-free rat plasma spiked with colistin (LLOQ) and IS (colistin concentrations of 78 ng/ml and 353 ng/ml for colistin A and colistin B, respectively).

**Figure 7:**
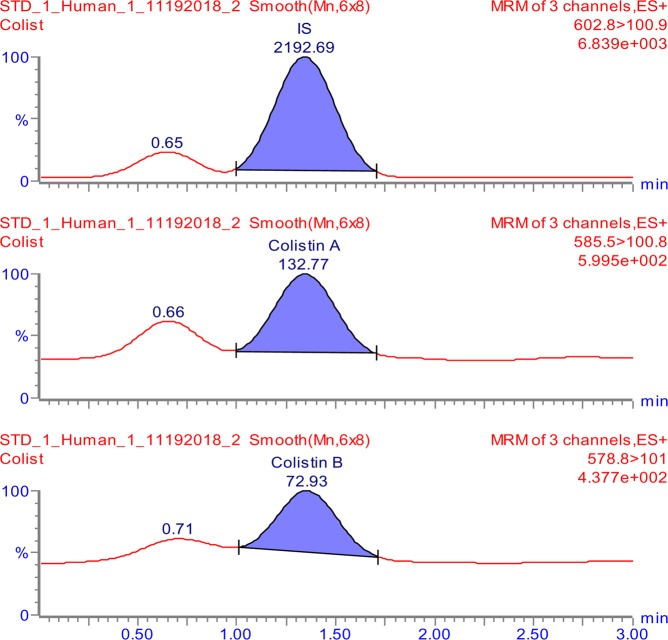
Typical LC-MS/MS-MRM chromatograms of a drug-free human plasma spiked with colistin and IS [colistin concentrations of 78 ng/ml and 353 ng/ml for colistin A and colistin B, respectively (LLOQ)].

**Figure 8:**
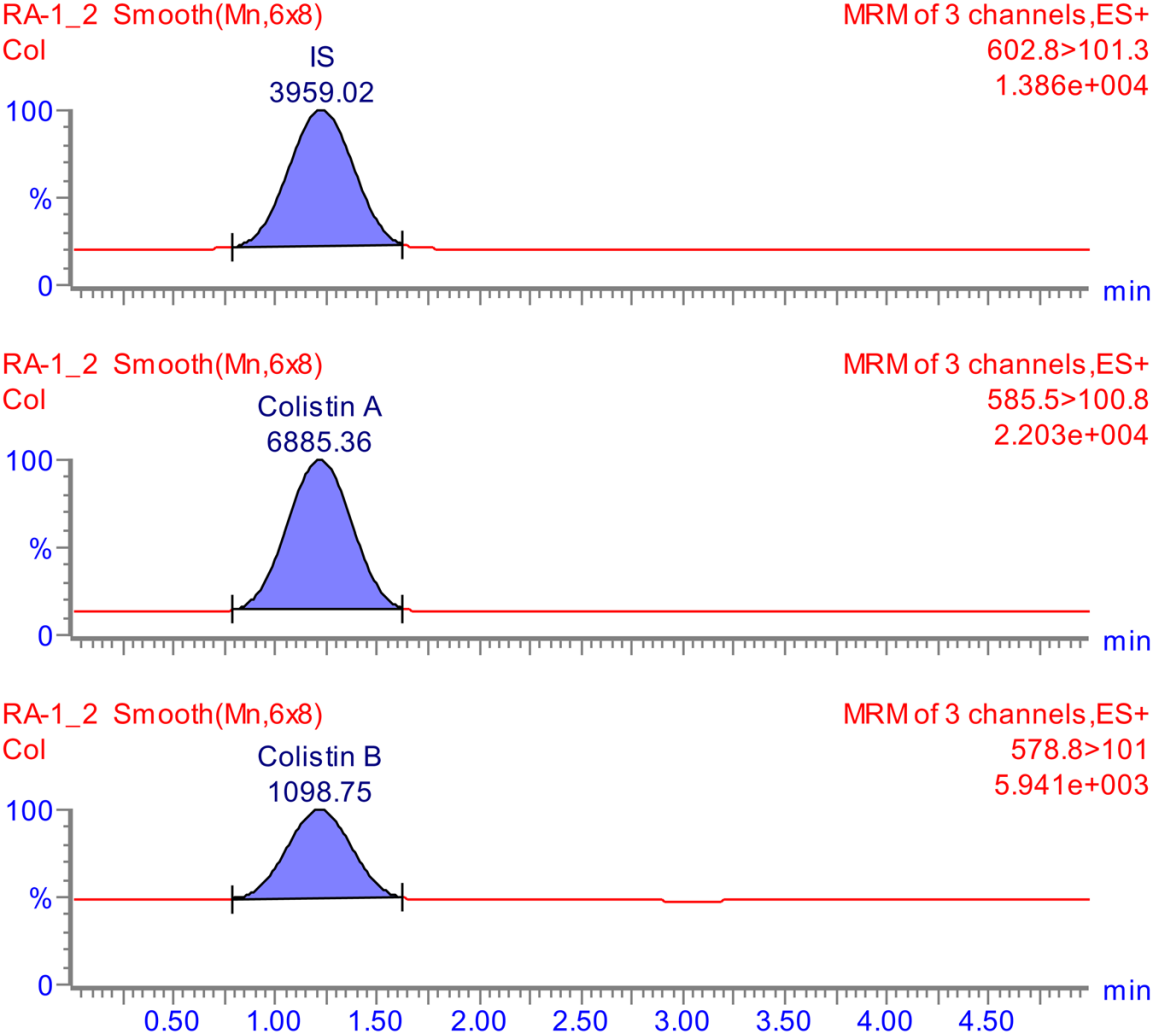
Typical LC-MS/MS-MRM chromatograms of a rat plasma sample collected 30 min following the IV administration of a dose of 15 mg/kg of colistin. (The calculated concentrations of colistin A and colistin B are 4105.8 and 2636.8 ng/ml, respectively).

The tuning parameters were optimized for both colistin A and colistin B using ESI + interface. The quantitation was carried out employing MRM mode. A mobile phase consisting of 30% (*v/v*) acetonitrile, 70% (*v/v*) water and 0.1% (*v/v*) formic acid was selected as the optimal mobile phase since it gave better formation of precursors and product ions than other compositions attempted. The column that provided the best peak shape and separation of analytes was Zorbax eclipse plus C_18_, rapid resolution, high definition (1.8 µm, 2.1 mm i.d. x 50 mm) (Agilent Technologies, Santa Clara, U.S.A.) with a flow rate of 0.2 mL/min. Figure 2(a) shows full scan mass spectra of the parent ions of colistin A at *m/z* 585.5 and colistin B at *m/z* 578.8 whereas, Fig. 2(b) illustrates the full scan mass spectra of precursor/product ions of the colistin A (*m/z* 585.5 > 100.8) while, Fig. 2(c) demonstrates the mass spectra of the precursor/product ions of the colistin B (*m/z* 578.8 > 101). The MS and MS-MS spectra of the IS (*m/z* 602.8 > 101.3) are shown in Fig. 3(a,b), respectively. Figures 4 and 5 demonstrate typical chromatograms of a drug-free rat plasma and a drug-free human plasma, respectively. The figures demonstrate lack of interfering endogenous compounds in the chromatograms of drug-free rat and human plasma samples. Spiking of the drug-free rat plasma with colistin and IS resulted in the MRM chromatogram depicted in Fig. 6. When a drug-free human plasma was spiked with colistin, the resulted MRM chromatograms are shown in Fig. 7. Moreover, Fig. 8 shows a typical plasma sample collected from a rat 30 min post-administration of 15 mg/kg of intravenous colistin sulfate.

### Method validation

**Figure 9:**
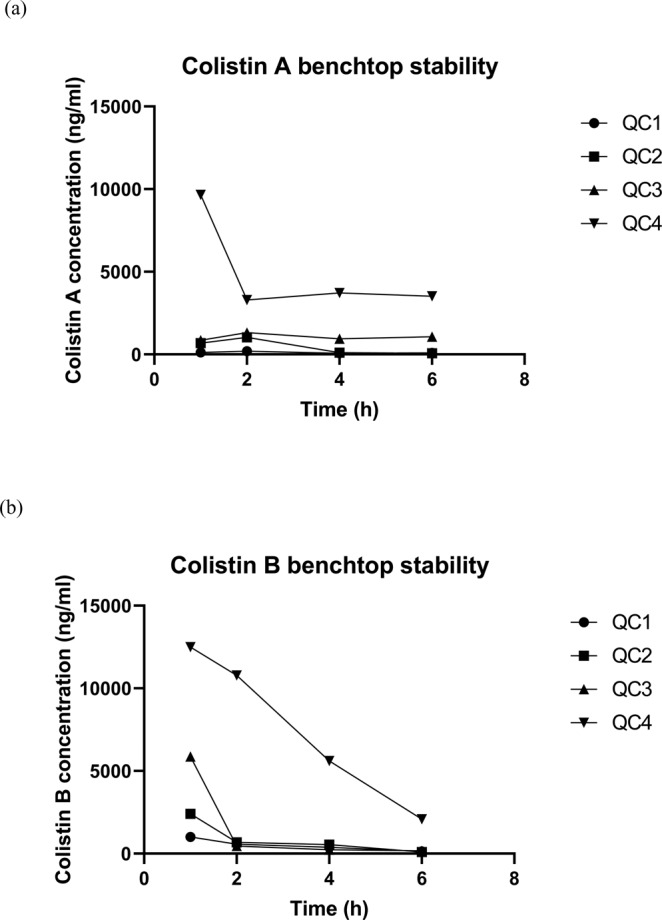
The benchtop stability study of (**a**) colistin A and (**b**) colistin B.

The method was validated over a colistin concentration range of 0.5–20 μg/mL. The calibration curves showed good linearity and reproducibility over the studied range (Table 1). The linear regression equation was: y = 0.27534 + 0.00087×(*n* = 10) for colistin A and y = 0.04124 + 0.00023x for colistin B, where y is the peak area ratio of colistin to the IS and x represents colistin concentration. The LLOQ of colistin was 0.5 µg/mL and its resultant signal was compared to that of a blank sample as described in Table 2. Intra-run precision (RSD%) at four different concentrations (234, 546, 1170 and 2730 ng/ml) of colistin A ranged between 2.7 and 8.4% (Table 3) whereas, the inter-run precision ranged between 7.8 and 14.0% (Table 4). The intra-run precision (RSD%) of colistin B at four different concentrations (1059, 2471, 5295 and 12355 ng/ml) ranged between 2.1 and 5.5% (Table 3) whereas, the inter-run precision ranged between 7.4 and 14.4% (Table 4). The intra-run accuracy (bias) for colistin A varied from −11.2 to −3.1% (Table 3) and from −5.1 to 4.4% for colistin B (Table 3). On the other hand, the inter-run accuracy (bias) for colistin A varied from −6.3 to 5.3% (Table 4) and from −3.8 to 11.0% for colistin B (Table 4). On the other hand, the data of colistin stability in rat plasma at different experimental conditions including freeze-thaw, long-term storage (−70 °C), benchtop stability and autosampler stability are presented in Table 5. Colistin showed acceptable freeze-thaw stability, long-term stability as well as autosampler stability (Table 5). However, it was not stable at room temperature (benchtop stability) when assessed for short-term (stored for 6 hours at room temperature), (Table 5 and Fig. 9). The matrix effect was evaluated and presented in Table 6. Furthermore, the carry-over study demonstrated lack of carry-over effect.

**Table 1:** Calibration curves summary*.

Colistin A				Colistin B			
No.	slope	intercept	r²	No.	slope	intercept	r²
1	0.0009	0.29887	0.99971	1	0.00023	0.029	0.99959
2	0.00086	0.32226	0.99879	2	0.00022	0.05733	0.99977
3	0.00082	0.29853	0.99643	3	0.00023	0.05291	0.99927
4	0.0009	0.27555	0.99718	4	0.00024	0.03402	0.99878
5	0.00084	0.26573	0.99825	5	0.00022	0.0313	0.99896
6	0.00084	0.24698	0.99781	6	0.00024	0.04002	0.99815
7	0.00083	0.27589	0.99631	7	0.00025	0.03275	0.99868
8	0.00086	0.29812	0.99736	8	0.00024	0.03887	0.99829
9	0.00091	0.26842	0.9977	9	0.00024	0.061	0.99592
10	0.00094	0.20304	0.99699	10	0.00023	0.03523	0.99798
**Mean**	0.00087	0.27534	0.99765	**Mean**	0.00023	0.04124	0.99854
**S.D**.	3.9E-05	0.03331	0.00105	**S.D**.	1.2E-05	0.01155	0.00109
**R.S.D%**	4.46725	12.0965	0.10543	**R.S.D%**	5.29019	28.0148	0.10965

*Studied within 20 days.

**Table 2:** LLOQ for determination of colistin in rat plasma by LC-MS/MS.

Standard 1	Colistin A	Colistin B
**Nominal Concentration (ng/ml)**	**78**	**353**
run 1	75.4	385.8
run2	88.7	342.7
run3	91.4	292
run4	74.1	322.6
**Mean**	82.4	335.8
**SD**	8.91777	39.3
**R.S.D %**	10.82	11.71
**Bias%**	5.64	−4.88

**Table 3:** Intra-run precision and accuracy for determination of colistin in rat plasma samples by LC-MS/MS (n = 6).

Nominal concentration (ng/ml)	Found (Mean ± S.D) (ng/ml)	R.S.D%	Bias%*
**Colistin A**			
234	214.70 ± 18.02	8.4	−8.25
546	485.08 ± 39.46	8.14	−11.16
1170	1070.19 ± 32.56	3.04	−8.53
2730	2645.95 ± 71.76	2.71	−3.08
**Colistin B**			
1059	1105.66 ± 55.18	4.99	4.41
2471	2372.24 ± 130.12	5.49	−4
5295	5027.08 ± 150.96	3	−5.06
12355	12044.99 ± 256.08	2.13	−2.51

**Table 4:** Inter-run precision and accuracy for determination of colistin in rat plasma samples by LC-MS/MS (n = 6).

Nominal concentration (ng/ml)	Found (mean ± S.D) (ng/ml)	R.S.D%	Bias%*
**Colistin A**			
234	246.395 ± 34.53	14.02	5.297
546	525.72 ± 56.89	10.82	−3.72
1170	1148.08 ± 89.73	7.82	−1.87
2730	2558.47 ± 219.69	8.59	−6.28
**Colistin B**			
1059	1156.54 ± 166.78	14.42	9.21
2471	2743.61 ± 338.11	12.32	11.03
5295	5228.54 ± 385.68	7.38	−1.26
12355	11890.26 ± 1017.37	8.56	−3.76

Precision and accuracy were determined from 6 different runs.

S.D: standard deviation.

RSD: relative standard deviation.

RSD (%) = (SD/Mean) *100.

Bias (%) = (mean of measured concentration-nominal concentration/nominal concentration) *100.

**Table 5:** Summary of colistin stability study in rat plasma samples by LC-MS/MS method.

Freeze-thaw (−70 °C to 25 °C after 24 hours)				
**Colistin A**				
**Nominal Concentration (ng/ml)**	**234**	**546**	**1170**	**2730**
Found (Mean ± S.D)(ng/ml) (n = 4)	265.4 ± 35.10	615.27 ± 30.29	1208.93 ± 77.51	2551.83 ± 177.97
R.S.D%	13.23	4.91	6.41	6.97
Bias%*	13.42	12.69	3.33	−6.53
**Colistin B**				
**Nominal Concentration (ng/ml)**	**1059**	**2471**	**5295**	**12355**
Found (Mean ± S.D)(ng/ml)(n = 4)	1188.48 ± 129.25	2715.48 ± 154.87	5136.5 ± 231.26	11293.18 ± 556.44
R.S.D%	10.88	5.70	4.50	4.93
Bias%*	12.23	9.89	−2.99	−8.60
**Long-term stability at −70 °C (20 days)**				
**Colistin A**				
**Nominal Concentration (ng/ml)**	**234**	**546**	**1170**	**2730**
Found (Mean ± S.D)(ng/ml) (n = 5)	237.24 ± 5.64	520.76 ± 19.46	1134.44 ± 26.11	2719 ± 50.20
R.S.D%	2.38	3.74	2.30	1.85
Bias%*	1.39	−4.62	−3.04	−0.40
**Colistin B**				
**Nominal Concentration (ng/ml)**	**1059**	**2471**	**5295**	**12355**
Found (Mean ± S.D)(ng/ml)(n = 5)	1073 ± 60.43	2457.56 ± 75.91	5280.04 ± 83.05	12524 ± 346.18
R.S.D%	5.63	3.19	1.57	2.76
Bias%*	1.32	−0.54	−0.28	1.37
**Benchtop Stability Study (25 °C for 6 hours)**				
**Colistin A**				
**Nominal concentration (ng/ml)**	**234**	**546**	**1170**	**2730**
Found (Mean ± S.D)(ng/ml) (n = 4)	120.275 ± 50.32	592.47 ± 488.56	1047.6 ± 202.94	5045 ± 3076.10
R.S.D%	41.84	82.46	19.37	60.97
Bias%*	−48.6	8.51	−10.46	84.8
**Colistin B**				
**Nominal concentration (ng/ml)**	**1059**	**2471**	**5295**	**12355**
Found (Mean ± S.D)(ng/ml)(n = 4)	518.72 ± 367.93	929 ± 1012.78	1684.55 ± 2793.21	7736.23 ± 4775.30
R.S.D%	70.93	109.02	165.81	61.73
Bias%*	−51.02	−62.40	−68.19	−37.38
**Autosampler stability study (4 °C for 24 hours)**				
**Colistin A**				
**Nominal concentration (ng/ml)**	**234**	**546**	**1170**	**2730**
**Found**				
(Mean ± S.D)(ng/ml) (n = 4)	231.18 ± 6.22	516.95 ± 53.28	1034.9 ± 38.64	2917.93 ± 341.07
R.S.D%	2.69	10.31	3.73	11.69
Bias%*	−1.21	−5.32	−11.55	6.88
**Colistin B**				
**Nominal concentration (ng/ml)**	**1059**	**2471**	**5295**	**12355**
Found (Mean ± S.D)(ng/ml)(n = 4)	1060.60 ± 108.61	2460.85 ± 115.75	4749.75 ± 386.29	12863.28 ± 1546.10
R.S.D%	10.24	4.7	8.13	12.02
Bias%*	0.15	−0.41	−10.30	4.11

SD: standard deviation.

RSD: relative standard deviation.

RSD (%) = (SD/Mean) *100.

Bias (%) = (mean of measured conc - nominal conc/nominal conc) *100.

**Table 6:** Matrix effect (ME) data for colistin A, colistin B and IS in six different rat plasma using LC-MS/MS method.

Nominal concentration (ng/ml)	Colistin A
	**Matrix effect (%)**
234	95.19
546	98.97
1170	103.22
2730	102.78
**Nominal concentration (ng/mL)**	**Colistin B**
	**Matrix effect (%)**
1059	105.42
2471	104.75
5295	103.45
12355	103.63
**Nominal concentration (µg/mL)**	**IS**
	**Matrix effect (%)**
500	95.19
500	105.35
500	105.86
500	105.48

The findings of incurred sample re-analysis (ISR) by using the present method demonstrated that the results were within the acceptable ranges (ranged between −19.3 to +18.6 and between −9.8 to 19.6 for colistin A and colistin B, respectively).

### Pharmacokinetic study

**Figure 10:**
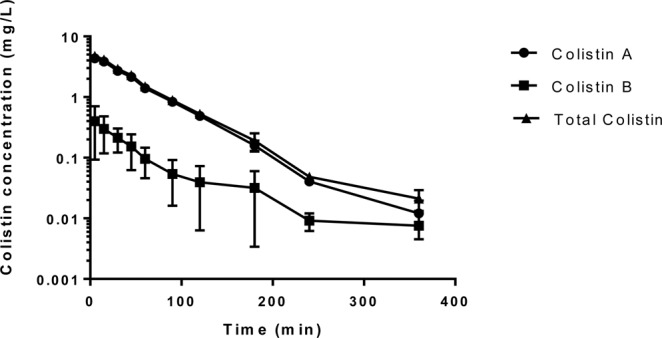
Mean (± SD) plasma concentration-time profiles of colistin A, colistin B and total colistin in rat plasma (n = 8).

Individual pharmacokinetic profiles of colistin in rats demonstrated pronounced inter-individual variabilities within the same group of animals. The mean (±SEM) plasma concentration-time profiles of colistin A and colistin B in rats (n = 8) is depicted in Fig. 10. Hematocrit levels were simultaneously measured for each rat blood sample collected for pharmacokinetic purposes. The mean (±SD) pharmacokinetic parameters of colistin in rats are presented in Table 7.

**Table 7:** Mean (± S.D) pharmacokinetic parameters of colistin in rats (n = 8).

Pharmacokinetic Parameter	Mean ± S.D.
t_1/2_ (min)	64.50 ± 36.30
AUC_0-∞_ (µg.min/ml)	631.37 ± 246.71
V_d_ (L/Kg)	2.45 ± 1.29
CL (mL/min/Kg)	25.53 ± 10.60

t_1/2_: half life.

AUC_0-∞_: Area under the plasma concentration-time curve.

V_d_: Volume of distribution.

CL: Total body clearance.

### LC-MS/MS analysis conditions

Tandem mass spectrometry (LC-MS/MS) analytical technique is a robust method that is used to analyze various types of compounds in biological fluids as well as applying it in pharmacokinetic studies and therapeutic drug monitoring. Low interferences with co-eluting compounds and high sensitivity are some of the reasons that expand its use worldwide.

An ideal IS usually has high similarity with the chemical structure of the analyte of interest, well resolved and doesn’t overlap with other peaks, is stable for a reasonable time and has similar physico-chemical properties as the analyte of interest. Stable isotope-labeled analogues are most commonly used as ideal internal standards in tandem mass spectrometry. However, they are costly and not widely available^38^. In the present study, PMB was chosen as an IS because of its high similarity in physico-chemical properties with colistin. In addition, it is cheap, and readily available^39^.

Several columns were attempted and we found out that the most suitable and appropriate column was Zorbax eclipse plus C_18_, rapid resolution, high definition (1.8 µm, 2.1 mm i.d. x 50 mm) (Agilent Technologies, Santa Clara, U.S.A.). Also, a number of mobile phases were tried and the mobile phase consisting of acetonitrile:water:formic acid (30:70:0.1 *v/v/v*) resulted in optimal conditions for separation as well as enhancement of the formation of colistin and IS ions. The chromatographic conditions were optimized to give the maximum response, minimum baseline noise with best separation and short run time (2 min).

Quantitation of colistin was achieved using ESI + interface employing MRM transitions at *m/z* 585.5 > 100.8 and *m/z* 578.8 > 101 for colistin A and colistin B, respectively whereas the MRM transition of the IS was at *m/z* 602.8 > 101.3. The standards and quality control samples concentrations were selected based on the therapeutic range of colistin. The expected colistin serum levels according to the antimicrobial reference laboratory assay service are 5–15 mg/L and 2–4 mg/L for the peak and tough concentrations, respectively^40^. However, Markou and colleagues^41^ found that colistin had a maximum steady state concentration of 2.93 ± 1.24 mg/L and a minimum concentration of 1.03 ± 0.44 mg/L^41^. Extracting the biological sample is an essential step in the analysis process as it isolates the analyte of interest and removes other compounds and impurities present in the biological sample. In the present method, several extraction techniques were attempted such as liquid-liquid extraction, protein precipitation and solid phase extraction (SPE). Two steps of plasma sample extraction procedure were selected which consisted of protein precipitation followed by an SPE procedure as they yielded better sensitivity and free of co-eluting endogenous compounds. A solution of 10% TCA and methanol at a ratio of 50:50 (*v/v*) was chosen to extract colistin and IS from rat and human plasma samples because of its high extraction efficiency.

### Method validation parameters

The linearity of the method was investigated and the calibration curves of colistin (n = 10) exhibited favorable linearity over the selected range of concentrations (0.5–20 μg/ml). The calibration curves were consistent and reproducible (Table 1). The LLOQ response was more than 5 times that of a drug-free plasma sample (Table 2). The intra-run and inter-run accuracy and precision for quantification of colistin A and colistin B in rat plasma samples at four different QC concentrations are presented in Tables 3 and 4, respectively. The data demonstrated that the intra-run accuracy (bias) for colistin A and colistin B ranged between −11.2 to −3.1% and −5.1 to 4.4%, respectively. However, the inter-run accuracy (bias) for colistin A and colistin B ranged between −6.3 to 5.3% and −3.8 to 11.0%, respectively. On the other hand, the intra- and inter-run precision (RSD%) for colistin A were in the range of 2.7 to 8.4% and 7.8 to 14.0%, respectively. Moreover, the intra- and inter-run precision (RSD%) for colistin B were in the range of 2.1 to 5.5% and 7.4 to 14.4%, respectively. The results demonstrated acceptable bioanalytical assay accuracy and precision parameters. The selectivity of the present assay method was evaluated and the resulting data exhibited lack of endogenous interfering peaks at the retention times of colistin A, colistin B and IS. In addition, colistin data demonstrated acceptable stability in plasma samples after five freeze-thaw cycles (−70 °C to room temperature) because the resulting concentrations were within 15% of the nominal ones. For evaluation of a long-term stability of colistin, a set of frozen (−70 °C) QC samples were extracted and analyzed. The results showed that the drug was stable in plasma samples for at least 3 weeks when kept frozen at −70 °C (Table 5). Colistin also demonstrated acceptable stability in the autosampler environment (4 °C) since the result at each level was within 15% of the nominal concentration (Table 5). However, bench-top stability exhibited variability in the results when colistin plasma samples were analyzed after storing at room temperature (25 °C) for 6 hours indicating in-stability of colistin on benchtop so, the samples need to be stored immediately in the autosampler (4 °C) prior to injection into the LC-MS/MS system (Table 5 and Fig. 9). The assessment of matrix effect on colistin plasma samples demonstrated lack of significant ion suppression or ion enhancement on colistin (Table 6). In other words, the presence of colistin in plasma and its components (such as phospholipids, serum proteins and anticoagulants) did not hinder or enhance the formation of the analyte’s detectable ions. Therefore, the matrix did not affect the sensitivity or selectivity of the present method. On the other hand, colistin demonstrated lack of carryover effect when injecting the drug-free plasma samples post to the upper limit of quantification (ULOQ). Thus, injecting colistin samples containing various concentrations would not be a problem and no precautions should be undertaken regarding this matter. In addition, the findings of incurred sample re-analysis (ISR) by using the present method demonstrated acceptable results.

### Pharmacokinetic parameters of colistin

Following the validation of the present analytical method, the method was applied to estimate the pharmacokinetic profile of colistin in experimental rats. Choosing the animal species to perform the pharmacokinetic study was based on the similarity of colistin pharmacokinetic profile between animal species and humans. In humans, it was found that colistin has a t_1/2_ of 180 min, V_d_ of 12.4 L^27^ and an apparent total body clearance (CL) of 3.6 L/min^42^. The species that has the closest pharmacokinetic profile to humans are rats^43–45^. In rats, colistin has a t_1/2_ of 74.6 ± 13.2 min, a V_d_ of 496 ± 60 ml/kg and CL_R_ of 5.2 ± 0.4 ml/min/kg^43^. The present study was conducted in 8 rats with an average age of 21 weeks ± 3 days which represents the age of social maturity in humans (around 33 years old)^46^. Hematocrit test was also performed since it is essential and it measures the red blood cells volume in blood compared to the total blood volume. The test is dependent on the plasma volume and reflects whether the body of the rat is hypovolemic, normovolemic or hypervolemic. This ensures that the colistin concentration results reflected the actual drug levels and not due to alterations in the volume of the blood^47^.

The pharmacokinetic profile of colistin is not yet fully understood, the appropriate dose of colistin is controversial and therapeutic drug monitoring (TDM) of colistin is not widely performed^14,48^. Therefore, investigating the pharmacokinetic profile of colistin becomes an area of interest for researchers^43–45,49^. In essence, TDM is principally important in patients receiving colistin because of its side effects^50^. In addition, the need to optimize colistin dose in various clinical cases renders TDM of colistin an essential tool^51^. Furthermore, monitoring of colistin levels may result in less intracranial complications because of the improvement in dosing levels in patients receiving colistin intrathecally^52^. TDM of colistin is of high impact in patients with complicated renal function^53^. In this regard, it has been reported that TDM of colistin is important in septic patients^54^. Therefore, TDM is an essential part of colistin treatment as it leads to safer and more effective therapeutic regimens^55^. It also helps in managing the common side effects associated with the use of colistin such as nephrotoxicity and neurotoxicity^55^.

In the present study, substantial variations in concentrations values among the rats were observed 5 min following IV dose of 15 mg/kg colistin. This is because of a distribution phase that occurs before the decline in the colistin concentration^43^. This finding is in agreement with previous reports^43,44,49^. In the present study, we observed that colistin concentration declined rapidly over 180–240 min which is consistent with earlier findings^45,49^. The total body clearance (CL) of colistin had a mean (±SD) value of 25.53 ± 10.6 ml/min/kg which is comparable to that reported by other investigators (11.7 ± 1.8 ml/min/kg)^44^. Sivanesan and co-workers^45^ reported the CL values of colistin A and colistin B separately, where colistin A CL was 2.75 ± 0.54 mL/min/kg and colistin B CL was 3.05 ± 0.62 mL/min/kg^41^. The mean (±SD) AUC_0-_ _∞_ value for eight rats was 631.4 ± 246.7 μg.min/ml (Range: 263.30 to 943.63 μg.min/ml), Table 7. The reported mean (± S.D.) value of AUC_0-_ _∞_ for colistin by other investigators was 2.6 ± 0.47 mg·h/L^44^.

The t_1/2_ of colistin in the present study was 64.50 ± 36.30 min which is consistent with the t_1/2_ reported earlier and it was 74.6 ± 13.2 min^43^. In another study, the t_1/2_ of colistin was reported as 55.7 ± 19.3 min^44^. In the dose ranging study, the t_1/2_ of colistin at the same dose used in this project (15 mg/kg) was 32.4 ± 5.0 min^49^. This parameter was also investigated in the major components of colistin in rats and was found that the t_1/2_ of colistin A was 82.0 ± 30.9 min while for colistin B it was 91.3 ± 20.5 min^45^. In the present study, the V_d_ of colistin was 2.45 ± 1.30 L/kg in contrast to an earlier report which was reported as 0.50 ± 0.06 L/kg^43^. In addition, we observed a substantial variation in the pharmacokinetic profile of colistin in each research group and this could be due to the significant inter-individual variability in the rate of the conversion of the prodrug to colistin *in vivo*. Furthermore, this might be due to the competitive disposition pathways for CMS in addition to the batch to batch variability and the complex nature of CMS^56^. On the other hand, we observed in the present study that hematocrit levels did not deviate by more than 5% in each rat. The consistency of hematocrit levels indicates that the change in the colistin levels was due to the actual change in the drug levels and not due to other factors such as hypovolemia. Hematocrit is important because it indicates the level of bleeding in the animal^57^, the effect of dilution on blood sampling^58^, helps in studying the plasma volume and blood anemia^59,60^. In other words, hematocrit was utilized in this study as one of the tests to make sure that the rats did not suffer from acute anemia due to blood loss and secondly the blood was not diluted and the blood levels and pharmacokinetic parameters of colistin were factual and valid.

The present method demonstrated many advantages such as short run time which is beneficial for high throughput large number of samples analysis, employing small sample volume (100 µL) which is appropriate particularly for pediatric patients, using MRM mode instead of SIM which is more specific and selective than the latter mode, reasonable LLOQ which is appropriate for TDM as well as optimization of colistin dosage regimen and also optimal for pharmacokinetic studies of the drug.

## Conclusions

The data presented in this study verified that colistin could be quantified with a rapid, specific, precise, and fully validated tandem mass spectrometric assay method. The method was applied in exploring the pharmacokinetic profile of colistin in rats. The present method is proposed to be of value in TDM for patients on therapeutic regimens involving colistin. However, the method needs to be fully validated in human plasma before employing it in humans for TDM purposes. This is of high value since TDM of colistin in patients will lead to a better utilization of colistin and minimization of its side effects and toxicity.
